# Sézary syndrome mimicking Steven–Johnson syndrome: A case report

**DOI:** 10.1097/MD.0000000000041080

**Published:** 2024-12-27

**Authors:** Sheng-Wei Fan, Kuan-Ming Lai

**Affiliations:** a Department of Hematology/Oncology, Yuanlin Christian Hospital, Yuanlin, Taiwan; b Department of Hematology/Oncology and Center of Hemophilia, Changhua Christian Hospital, Changhua, Taiwan.

**Keywords:** cutaneous T cell lymphoma, mycosis fungoides, Sézary syndrome, Steven–Johnson syndrome, toxic epidermal necrolysis

## Abstract

**Rationale:**

Sézary syndrome is a rare and subtype of non-Hodgkin T cell lymphomas. It typically presents as papules, macules, nodules, or ulcers, frequently accompanied by lymphadenopathy and systemic symptoms. This study describes uncommon clinical and diagnostic features to distinguish Sézary syndrome from serious skin and mucous membrane disorders through a detailed case report and comprehensive literature review.

**Patient concerns/Diagnosis/Intervention/Outcomes:**

A 70-year-old female patient was diagnosed with progressive Sézary syndrome associated with extensive cutaneous scaly and ulcerative lesions after 5 cycles of anthracycline-based chemotherapy which is initially misleading to Steven–Johnson syndrome (SJS). SJS was clinically excluded due to no mucosal involvement. She received a single agent of steroid as a lymphoma salvage treatment due to poor performance and nutrition status. Facial and left arm skin biopsy revealed T lymphoma cells infiltrating the upper dermis. Unfortunately, she died due to progressive lymphoma disease and upper gastrointestinal bleeding.

**Lessons:**

Unlike mycosis fungoides, Sézary syndrome involves more than bathing suit areas of skin and it may present with scaly, ulceration, or even hemorrhagic bullae and Sézary cells in peripheral blood. Sometimes, it’s hard to differentiate from SJS or toxic epidermal necrolysis (TEN). Mucosa involvement is a crucial feature shown in patients with SJS/TEN.

## 1. Introduction

Cutaneous T-cell lymphomas (CTCLs) are a rare group of lymphomas that primarily affect the skin. The most common subtype of CTCL is mycosis fungoides (MF), which has an incidence of approximately 6 cases per million people per year in the United States, accounting for about 4% of all non-Hodgkin lymphoma cases.^[1]^ The incidence rate is lower among Asian countries. The peak age of presentation is around 60 years, with a males predominance.^[2]^ The second most common CTCL is Sézary syndrome (SS), which is characterized by distinctive erythrodermic lesions, leukemic involvement of clonal malignant T cells, and multiple lymphadenopathies. SS typically presents with more aggressive clinical features and a worse prognosis than MF.

Diagnosing MF/SS can be challenging, as the skin lesions often resemble inflammatory skin conditions such as eczema or psoriasis, especially in the early stages. Later stages may be misdiagnosed as Steven–Johnson syndrome (SJS), toxic epidermal necrolysis (TEN), or cutaneous infections. Given that the treatment for MF/SS differs significantly from that of other differential diagnoses, early detection and further diagnostic evaluation are crucial for effective management.

## 2. Case presentation

**Figure 1.**
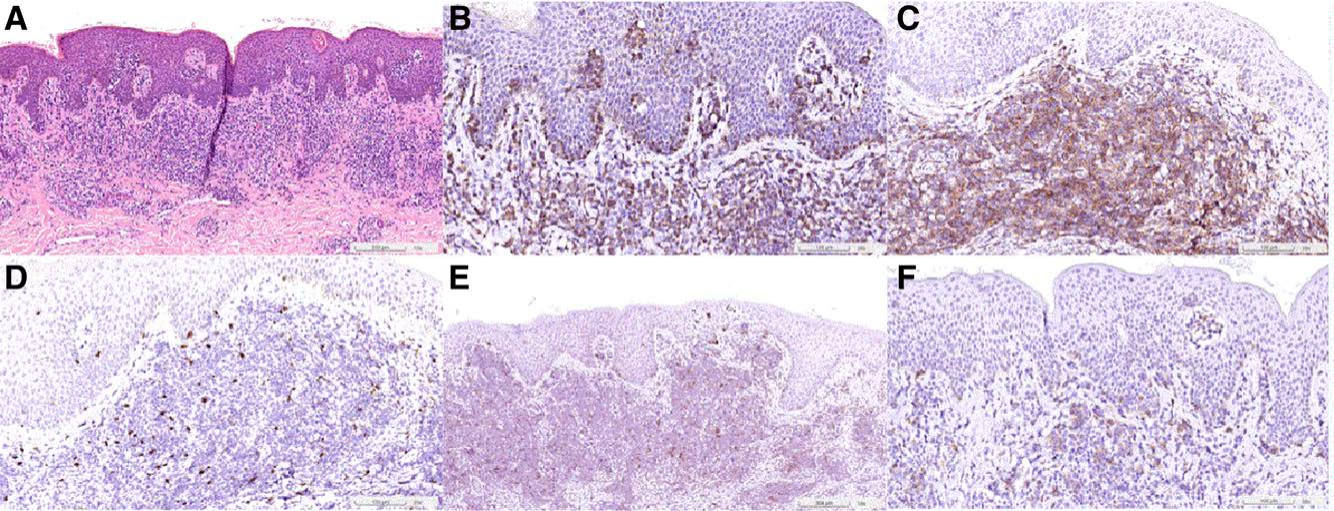
Microscopically, it shows small- to medium-sized pleomorphic and epidermotropic lymphoid cells in the upper dermis. The intraepidermal nest of atypical lymphocytes is commonly known as “Pautrier’s microabscess.” [HE stain, 100×] (A). Almost all lymphoid cells were positive for CD3 (B) [200×] and CD4 (C) [200×]; negative for CD8 (D) [200×], CD25 (E) [100×], and CD30 (F) [200×].

A 70-year-old female patient with a medical history of hypertension and hyperlipidemia was diagnosed with Sézary syndrome, stage IV, in November 2021. Her initial presentation included multiple erythematous papular and macular skin lesions and peripheral blood analysis revealed Sézary cells with a high CD4/CD8 ratio, as determined by flow cytometry. A skin biopsy and immunohistochemical analysis of skin lesions showed predominant CD3 without CD20 expression, with more CD4-positive T cells than CD8-positive T cells among the CD3-positive atypical cells. The biopsy also revealed a lack of CD25 and CD30 expression (Fig. 1A–F). Bone marrow trephine biopsy revealed no lymphoma cells, and a positron emission tomography–computed tomography (PET/CT) scan revealed multiple enlarged lymph nodes with 2-Deoxy-2-[18F] fluoro-D-Glucose (FDG) uptake, consistent with malignant lymphoma. Laboratory tests in September 2023 revealed normal hemoglobin and platelet counts but elevated white blood cells and lactate dehydrogenase (LDH) levels. The patient was negative for human T-lymphotropic virus-1 (HTLV-1) and Epstein–Barr virus.

The patient received 5 courses of anthracycline-based chemotherapy starting in December 2022. A follow-up PET/CT scan in March 2023 revealed a partial response in the bilateral groin, but persistent nodular FDG hypermetabolism in the left axilla region. Additionally, skin lesions regressed and Sézary cells disappeared after treatment. However, treatment was discontinued due to an acute cerebral stroke in May 2023, and she resumed prednisolone monotherapy in June 2023 as her clinical condition improved.

**Figure 2.**
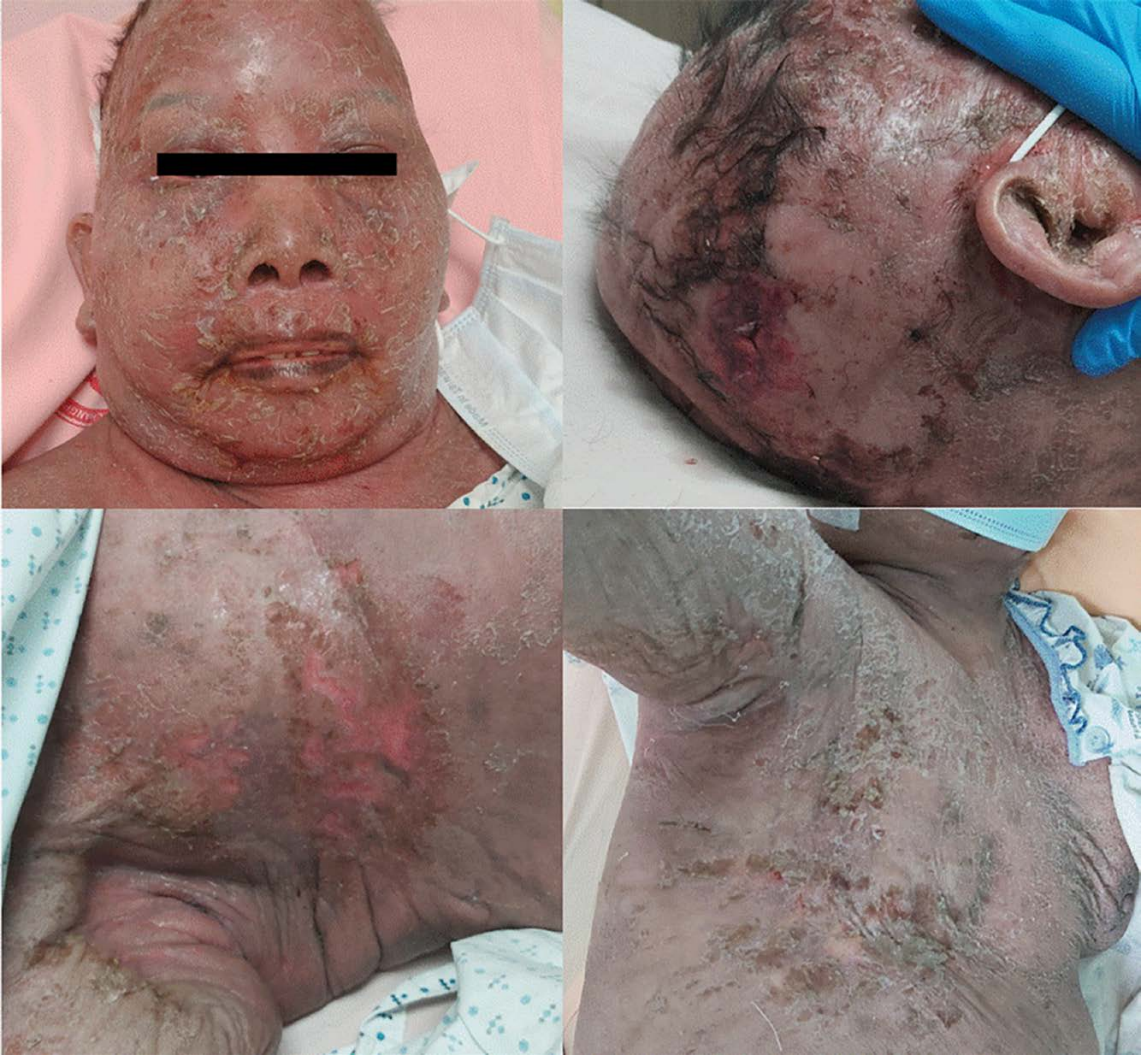
Extensive cutaneous scaly, protruding and ulcerative lesions over scalp, face, axilla and trunk.

**Figure 3.**
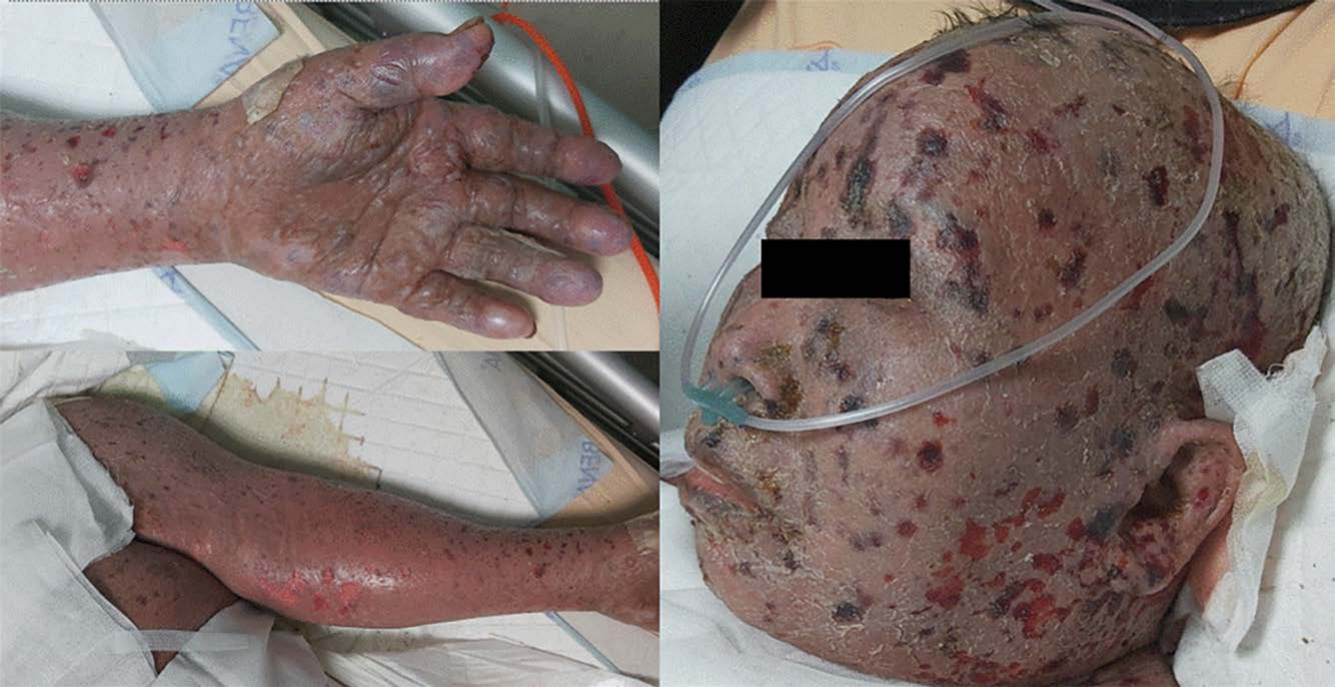
Hemorrhagic bullae formation and ulcerative lesions present over the face. Progressed erythema, ulcerative lesions, and bullae formation over left arm and palm.

In August 2023, the patient presented with a head injury and progressing thick scaling lesions around the face as well as several papular and macular lesions on the scalp. Multiple new erosive papules appeared over the right axilla, buttocks, and left arm, along with a small bullae formation. The trunk and extremities demonstrated generalized hyperpigmentation (Fig. 2). Mucosal surfaces in the oral, conjunctival, and genital areas were intact. She experienced no fever, chills, or allergic symptoms. Blood tests revealed leukocytosis with lymphocyte predominance and elevated LDH and uric acid levels. The liver and renal function were within normal limits. SJS was first excluded based on the absence of mucosal involvement. SS progressed with increasing skin involvement and atypical skin infection were favored. Methylprednisolone and antibiotics were administered after clinical suspicion. Two days later, a peripheral blood test revealed Sézary cells again, indicating disease progression. Left arm and face skin biopsy revealed predominant CD3 and CD4 expression T-cell lymphoma involvement. Several hemorrhagic bullae over the face (Fig. 3) was presented 1 week later. Unfortunately, she died due to progressive lymphoma disease and perforated peptic ulcer along with hemorrhagic shock. This study was approved by the Institutional Review Board of Changhua Christian Hospital (IRB number: 240423). Informed consent was also obtained from the patient for publication of this case report details.

## 3. Discussion

Cutaneous T lymphoma and peripheral T cell lymphomas (PTCLs) with skin involvement are 2 distinct diseases. Cutaneous T lymphoma is usually classified as MF and SS. MF, follows an indolent clinical course and is often confined to the skin. However, it may progress to enlarged lymphadenopathy or the leukemic status of lymphoma cells in which the disease progressed. MF is characterized by heterogeneous cutaneous features, from papules, macules to generalized erythroderma and even alopecia, erosions, and bullous formation. It is frequently characterized by progressive lesions that develop in a “bathing suit” distribution.^[3]^ An SS diagnosis is made when a high number of Sézary cells circulate in the peripheral blood especially in the presence of a cutaneous erythroderma occupying > 80% of the body surface area (BSA) and generalized lymphadenopathy. Extracutaneous sites or visceral organs can be seen in SS.^[4]^ PTCLs with skin involvement are often accompanied by lymphadenopathy, hepatosplenomegaly, or even bone marrow infiltration and usually have poor outcomes. Generally, PTCLs do not exhibit Sézary cells in peripheral blood.

Cutaneous T cell lymphoma reveals positive CD2, CD3,CD4,TCR-beta, characteristically lacking CD7, CD8, CD25 and CD30; and usually expressing PD1 by immunohistochemical stain.^[5]^ Immunophenotyping confirms that T-cell origin (CD3+ and CD4+) and lack of expression of CD7, CD30 are supportive of cutaneous T cell lymphoma.The loss of CD26 expression and positivity for KIR3DL2 (CD158k) has a high specificity for SS.^[6]^

SJS and TEN are characterized by extensive necrosis and detachment of the epidermis. TEN is defined as >30% of BSA lesions, and SJS is <10% of BSA. Mucous membranes are affected in > 90% of patients. Prodromal symptoms include conjunctivitis, fatigue, fever, and sore throat.^[7]^ SJS lesions typically begin with ill-defined, coalescing, erythematous macules, which evolve over the course of 1 to 2 days to develop dusky erythema, purpuric spots, atypical target lesions, and flaccid bullae. Extensive sheet-like detachment and erosions occur as the disease progresses. The Nikolsky sign is usually positive. The scalp is typically not affected, and painful erythema typically develops on the palms and soles. SJS/TEN mostly occurs 3 to 4 days after exposure to triggers.

The optimal therapy for cutaneous T lymphomas is determined by disease stage according to TMNB classification. For early-stage MF (stage I to IIA), skin direct therapy such as topical agents (e.g., corticosteroid, carmustine), and phototherapy (e.g., UVB, PUVA) are generally recommended. For the advanced stage of MF (stage IIB to IV) and SS, systemic therapy is often necessary.^[8]^

## 4. Conclusion

Cutaneous T cell lymphoma can masquerade as SJS or TEN, and differentiating them from clinical manifestation is difficult. Evaluation of skin lesion patterns, involved sites, clinical courses, and most importantly, mucosal intactness may provide hints to distinguish these diseases. However, skin biopsy remains the gold standard of definite diagnosis.

## Author contributions

**Conceptualization:** Sheng-Wei Fan.

**Project administration:** Sheng-Wei Fan.

**Resources:** Kuan-Ming Lai.

**Writing – original draft:** Sheng-Wei Fan.

**Writing – review & editing:** Sheng-Wei Fan, Kuan-Ming Lai.
